# Auditory Deficit as a Consequence Rather than Endophenotype of Specific Language Impairment: Electrophysiological Evidence

**DOI:** 10.1371/journal.pone.0035851

**Published:** 2012-05-09

**Authors:** D. V. M. Bishop, Mervyn J. Hardiman, Johanna G. Barry

**Affiliations:** Department of Experimental Psychology, University of Oxford, Oxford, United Kingdom; University College London, United Kingdom

## Abstract

**Background:**

Are developmental language disorders caused by poor auditory discrimination? This is a popular theory, but behavioural evidence has been inconclusive. Here we studied children with specific language impairment, measuring the brain’s electrophysiological response to sounds in a passive paradigm. We focused on the T-complex, an event-related peak that has different origins and developmental course from the well-known vertex response.

**Methods:**

We analysed auditory event-related potentials to tones and syllables from 16 children and 16 adolescents with specific language impairment who were compared with 32 typically-developing controls, matched for gender, IQ and age.

**Results:**

We replicated prior findings of significant reduction in Ta amplitude for both children and adolescents with specific language impairment, which was particularly marked for syllables. The topography of the T-complex to syllables indicated a less focal response in those with language impairments. To distinguish causal models, we considered correlations between size of the Ta response and measures of language and literacy in parents as well as children. The best-fitting model was one in which auditory deficit was a consequence rather than a cause of difficulties in phonological processing.

**Conclusions:**

The T-complex to syllables has abnormal size and topography in children with specific language impairment, but this is more likely to be a consequence rather than a cause of difficulties in phonological processing.

## Introduction

**Figure 1:**
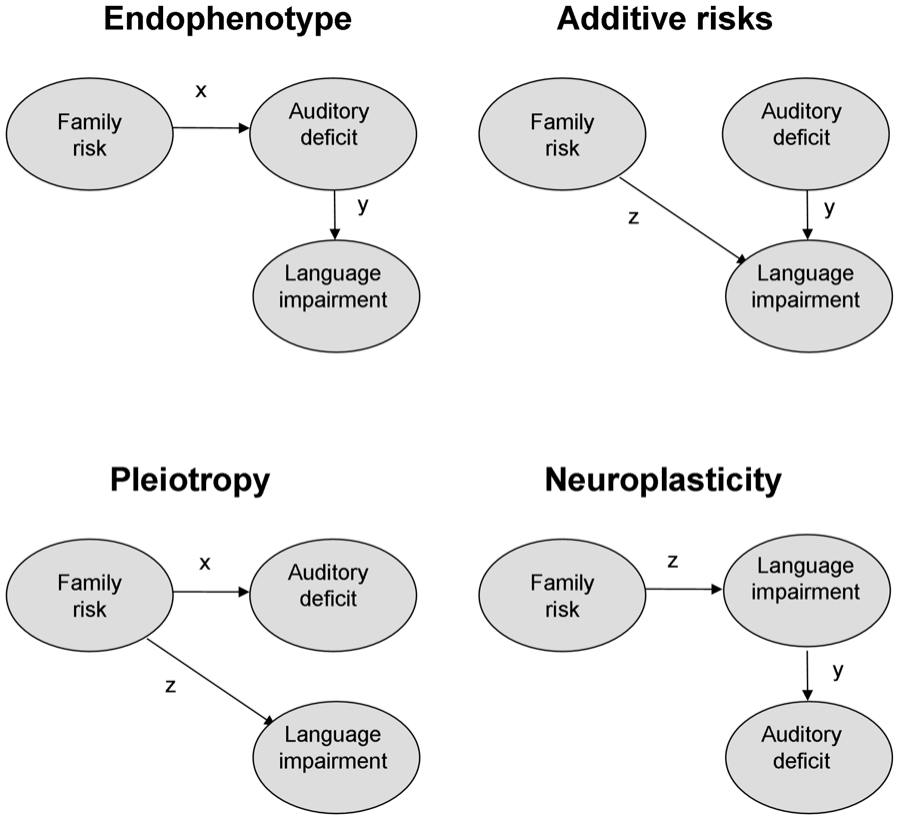
Causal models of the relationship between auditory deficit, language impairment and family risk for SLI.

Specific language impairment (SLI), also known as “developmental dysphasia”, is a heritable neurodevelopmental disorder that is diagnosed when a child has difficulties learning to produce and/or understand speech for no apparent reason [1]. One theoretical account of SLI regards oral language difficulties as a downstream consequence of low-level auditory perceptual limitations. Although such theories have a long history, debate continues as to whether SLI is essentially an auditory processing disorder, and if so, whether the auditory limitations are specific to speech [2]. Despite some 40 years of research it has been surprisingly difficult to resolve this issue. In part, this is because correlational data are poor at distinguishing causal models. All four models shown in Figure 1 predict an association between auditory deficit and language impairment. The Endophenotype model is often assumed to be the explanation if a correlation is found: this regards auditory deficit as a mediating factor between a genetic risk for SLI and overt language deficits. The Additive Risks model, on the other hand, regards auditory deficit as an additional risk factor that moderates a genetic risk for language impairment [3]. Another possibility is that the two deficits are independent consequences of a genetic risk variant, as shown in the Pleiotropy model. Finally, in the Neuroplasticity model, auditory deficit is the *consequence* of language impairment, with brain processing of sounds being affected by poor language skills. As will be argued below, progress can be made in distinguishing these causal models if we have measures of parental language as well as child auditory and language skills.

As well as logical problems, there are methodological difficulties inherent in this field. It is difficult to get reliable data from children using psychoacoustic procedures that involve long sequences of uninteresting stimuli. Electrophysiological methods have been proposed as an approach that could overcome this problem [4], but in practice it has been difficult to find solid, replicable effects that could be the basis for a clinical test [5]. One of the most popular neurophysiological indicators of auditory discrimination, the mismatch negativity (MMN) is useful in group comparisons, but insufficiently reliable or valid for clinical diagnosis of individuals [6].

Recently, attention has shifted from the MMN to measures based on the auditory event-related potential, obtained by averaging the waveform from electrodes placed on the scalp during an interval associated with stimulus presentation [7]–[10]. Most research has focused on the vertex response, an auditory event-related potential (AERP) with a distinctive pattern of peaks and troughs, which is sensitive to age and aspects of stimulus presentation [11]. AERPs recorded from temporal sites differ from the vertex response, being generated in secondary auditory cortex, and lateralised with a right-sided predominance [12]–[13]. Also, the T-complex has a different developmental trajectory to the vertex response as it matures earlier and remains relatively constant in form from puberty onwards [12]–[17].

The AERP recorded from temporal sites was first studied in detail by Wolpaw and Penry [18], who described a series of negative (∼90 ms), positive (∼110 ms) and negative (∼125 ms) peaks at temporal sites. They suggested that this was composed of the vertex N1–P2 complex originating from a wide area of cortex plus an additional wave in the 80–200 ms range produced within secondary auditory cortex. In order to reveal the additional wave, the N1–P2 response at the vertex (electrode Cz) was scaled to, and subtracted from, that recorded at temporal sites. The resultant waveform was labelled the T-complex, a positive wave at 105–110 ms (Ta) followed by a negative deflection at 150–160 ms (Tb). Subsequent researchers have retained the term T-complex but have not adopted the approach of subtracting the standardized vertex response. This seems appropriate given that there is little correlation between the response recorded at the midline electrodes and that recorded at temporal sites [13].

The amplitude and shape of the observed waveform at temporal sites will be influenced by the reference used. Wolpaw and Penry [18] used a non-cephalic reference, but others have used the nose, electrode FPz, or average of all electrodes. However, the latter approach is potentially problematic because of the large impact of the vertex response on the average, which, if subtracted, can lead to a waveform that is dominated by an inverted vertex response.

Recently, Shafer et al [19] reanalysed data from a series of ERP studies, and found differences in the T-complex (recorded with a nose reference) between children with SLI and matched typically-developing children. Group differences were most marked for the Ta positive peak, which occurs around 80–160 ms after the onset of an auditory signal. Among children with language impairments, 73% had poor T-complex measures, compared with only 13% of the typically-developing children. Shafer et al noted that this striking difference is consistent with some much older, neglected studies. Mason and Mellor [20] first described abnormal responses to a 1000 Hz tone at temporal sites in children with speech and language disorders. Their sample of children was not well-described, with no psychometric data on IQ or language, and this aspect of their results has not received much attention. Tonnquist-Uhlén [9] included the T-complex in an extensive study of auditory event-related potentials in 20 children with severe language impairments. She found that a substantial minority of children did not show a Ta component to a 500 Hz tone, compared with only one of twenty control children. However, psychometric data were not provided, and the EEG was pathological in nine of the language-impaired children and borderline in two. Only two children had a family history of language impairment. In this regard, the sample was rather different from that of most English-speaking samples with SLI, where there is typically high familiality and no obvious neurological abnormalities [21].

The results of Shafer et al [19] suggest that the auditory ERP at temporal sites may be a useful index for documenting abnormal brain responses to sounds in children with SLI in a context where there are no task demands beyond sitting passively while auditory stimuli occur. The current analysis aimed to replicate their findings for the Ta amplitude recorded at temporal sites, and to address further issues that are important for fully understanding the significance of this result, as follows:

- All of Shafer et al’s studies involved speech stimuli (vowels, nonword syllables, and words).The earlier studies of Mason and Mellor [20] and Tonnquist-Uhlén [9] suggested that deficiencies of the T-complex are also found for nonverbal stimuli (tones). In the current study, we compared responses recorded at temporal sites to syllables and tones.
- Another question concerns lateralisation of the T-complex. With monaural presentation, the T-complex is larger on the side contralateral to stimulation, but superimposed on this effect is an asymmetry that is evident even with binaural presentation, whereby the T-complex is larger on the right side [22]–[23]. This raises the question of whether this asymmetry develops normally in SLI, where atypical cerebral lateralisation for higher language functions has been observed [24]–[25].
- Where reduced amplitude of an AERP component is found, it is usually assumed that the brain is less sensitive to the signal. However, two other explanations need to be considered. First, the recording may simply be noisier than usual, masking the peak. Second the relevant component may be present, but in a different topographical location.
- If the T-complex is abnormal in children with SLI, this could represent delayed maturation, or a more persistent abnormality [26]–[27]. To distinguish these options, as well as assessing children of similar age to previous studies, we included a group of teenagers.
- Finally, the Ta component could be regarded as an endophenotype for heritable SLI (see Figure 1). For this to be the case, abnormality of Ta would need to meet two criteria: (i) it would have to be correlated with measures of language or literacy in children; (ii) it would need to be a mediating variable that could explain shared variance on language/literacy measures between affected children and their parents. Prior research has identified rather few correlations between electrophysiological measures and language or literacy skills, though in general the focus has been on MMN rather than the T-complex [5]. Where positive associations between behavioural measures and auditory ERPs (or magnetic counterparts) have been found, these have predominantly involved phonological processing [6] [8] [28], reading [29] or receptive language skills [7]. These behavioural measures were therefore selected as the focus of the current study.

For the purposes of this study, we used a dataset that was originally collected to examine mismatch responses to sounds in children and teenagers with SLI and their typically-developing (TD) controls [30]. We were able to replicate the finding of abnormal T-complex in children and teenagers with SLI, but a model of T-complex abnormality as an endophenotype did not give the best fit to the data.

## Materials and Methods

### Participants

Children (N = 16) and teenagers (N = 16) with SLI were recruited from special schools and classrooms for children with language or literacy problems. Inclusion criteria were a scaled score of 1.3 SD or more below average on at least two of eleven language measures (see below), a nonverbal IQ of 80 or above, and normal hearing for speech frequencies (500 to 4000 Hz). Typically-developing controls (N = 32) were matched on age and nonverbal IQ to the children with SLI. All had nonverbal IQs of 80 or above and passed a hearing screen. No child was selected purely on the basis of poor literacy scores. All participants had taken part in the study reported by Bishop et al [30], which gives further details. The study was approved by the Oxford Psychiatric Research Ethics Committee and parents of all participants gave written informed consent.

### Diagnostic Test Battery

Nonverbal ability was assessed using the two performance subscales of the Wechsler Abbreviated Scale of Intelligence [31]. The standardized instruments used to identify language impairment were: (i) A parent checklist giving a global index of communication problems - the Children’s Communication Checklist, version 2 [32]. (ii) A test of oral language comprehension: The Test for Reception of Grammar, version 2 (TROG-2) [33]; (iii) The Expression, Reception and Recall of Narrative Instrument (ERRNI) [34], which provides measures of mean length of utterance, story content, story recall, and story comprehension. (iv) The NEPSY [35], which provides measures of nonword repetition, sentence repetition and oromotor skills; (v) Reading of words and nonwords, assessed by The Test of Word Reading Efficiency (TOWRE) [36]. With the following exceptions, scores were converted to scaled scores according to test manuals: Tables of norms for NEPSY oromotor skills give conversion of raw scores to a five-point scale corresponding to centile ranges. For NEPSY nonword repetition, which was modified to be suitable for children speaking British English, we used our own norms to cover the full age range studied here. The measures used for causal analysis were nonword repetition, oromotor skills, nonword reading and TROG-2. Internal consistencies for British children were .83, .74 and .90 for the first three of these tests [37] and .88 for TROG-2 [33]. Estimation of structural model fit works best if measures are on a similar scale, so scores on relevant variables were converted to z-score relative to the standardisation norms, except for oromotor skills, where 3 was subtracted from the centile range score provided in the manual to give a range from −2 to 2.

### Measure of Familial Risk for SLI

A measure of family risk for SLI was created using data from Barry et al [38], who had administered a language battery to at least one parent of 60 of the 64 participants in this study. This battery included four of the measures given to children: NEPSY nonword repetition, Test for Reception of Grammar-E [39] and the Test of Word Reading Efficiency [36] and NEPSY oromotor sequences [35]. Scores on TROG-E and TOWRE were converted to z-scores using published test norms; for the NEPSY tests, where adult norms are lacking, we used our own normative data from a larger sample of parents of typically-developing children.

### Electrophysiological Methods

#### Stimuli

Stimuli are described in detail by Bishop et al. [40]. The analysis reported here was restricted to standard stimuli, i.e., a 1000 Hz tone and a modified natural speech token of the syllable “bah”. The stimuli had durations of 175 ms, were windowed at 15 ms, and were presented monaurally to the right ear at 86.5 dB SPL through sound-attenuating Sennheiser HD25-1 headphones.

#### Procedure

Tone and syllable stimuli were presented in separate blocks. Standards were presented on 70% of trials, with one of two other (deviant) stimuli occurring on 15% of trials in a quasi-random sequence. Deviant stimuli, which are not analysed here, were a 1030 Hz and 1200 Hz tone for the tone condition, and the syllables “bee” or “dah” for the speech condition. Stimulus onset asynchrony was 1 s. For each stimulus type there were two blocks each of 333 trials, making a total of 466 standards. Participants were seated in a sound-attenuated electrically-shielded booth, and they played Gameboy or watched a silent film during stimulus presentation.

#### EEG recording and data analysis

The EEG was recorded on a SynAmps or NuAmps NeuroScan system using Ag/AgCl sintered electrodes and a water-soluble conductive gel. Early pilot studies indicated no difference in the results obtained from the two recording systems, and the proportions of children from each group tested on each system did not differ significantly. An electrode cap was fitted according to the International 10–10 system to record from 28 sites: FC1, F7, FP1, FZ, FP2, F8, FC2, FT9, FC5, F3, FCZ, F4, FC6, FT10, T7, C3, CZ, C4, T8, CP5, P7, P3, PZ, P4, P8, CP6, M1, and M2. M1 or M2 was selected as reference electrode and ground was placed at AFZ. Electro-oculograms (EOG) were recorded from supra- and infra-orbital electrodes on the left eye and also from electrodes placed lateral to the left and right eyes. Impedances for all electrodes were kept below 8 kΩ. The EEG was recorded continuously on-line and stored for off-line processing. EEG data were digitised at 500 Hz and band-pass filtered (0.01–70 Hz for SynAmps; 0.1–70 Hz for NuAmps) and a 50 Hz notch filter was employed.

#### Offline analysis

The continuous EEG was epoched to give trials for all standard stimuli of 1000 ms duration, including 200 ms baseline. Subsequent data processing is described in detail by Bishop et al. (2011b). This was done for each participant separately for standard tone and syllable stimuli. An initial stage of artefact rejection was conducted to remove trials with extreme amplitudes (+/−350 µv), while retaining blinks. Blinks and other regular artefacts were then mathematically subtracted from the data using independent component analysis (ICA) (see [12]. Further artefact rejection was then applied with cutoff +/−75 µv.

Conventional analysis of the Ta component was conducted on the averaged AERP for each participant. Mean amplitude of Ta was measured at the temporal electrodes T7 and T8 over the time window 88–160 ms post signal onset, which was the interval used by Shafer et al [19] for this component. Mean amplitude rather than peak amplitude was used because of concerns that peaks are more affected by noise in the signal. Following Shafer et al [19], the Ta was measured relative to a baseline of 0–76 ms post stimulus onset. Internal consistency was computed by measuring Ta amplitude separately for odd and even epochs for each stimulus type and electrode. Correlations between Ta amplitudes for odd and even epochs were .78, .76, .85 and .88 for tones (T7 and T8) and syllables (T7 and T8) respectively. To ensure normal data for structural equation modeling, values that were more or less than 2 SD from the mean for that electrode and condition were censored, i.e. a value corresponding to ±2 SD was substituted, ensuring that skewness and kurtosis were nonsignificant for all Ta measures. This affected 5% of values. In addition, the noisiness of the signal at electrodes T7 and T8 was estimated using the standard deviation of the amplitude across time in the pre-stimulus period (−200 to 0 ms) of the waveform.

To visualise the topography of the auditory ERP the grand average waveforms for each group and stimulus combination were analysed to extract two independent components following the procedures adopted by Bishop et al [12]. This approach allows one to investigate topography of activity after extracting the P1–N2–P2 vertex response, which dominates the auditory ERP and usually emerges as the first component. In our data, the later N2 (peaking around 400 ms) was also substantial and a 2 Hz high-pass filter was first applied to minimize its impact on the auditory ERP. This filtering has minimal impact on the T-complex and N1–P2 complex, which occur earlier, and it ensures we do not have a component corresponding to N2 emerging as one of the first two components. The EEGlab routine ‘runica’ was then applied to each grand averaged waveform to extract two independent components after reducing the dimensionality of the data with principal components analysis. Polarity of components is arbitrary: for group comparisons, polarity of component 1 was made consistent with Fz, and polarity of component 2 was made consistent with T7.

### Analytic Approach

Our analysis focused on amplitude of the Ta component at T7 and T8, as this gave the clearest evidence of group differences in previous studies and we wished to use a priori statistical tests. The null hypothesis was that Ta amplitude would be similar in SLI and TD groups. Comparisons of quantitative AERP indices in SLI and TD were conducted using four-way analysis of variance (ANOVA) with stimulus (tone vs syllable) and side (T7 vs T8) as repeated measures, and clinical status (SLI vs TD) and age band (child vs teen) as between subjects variables. Topography of the AERP was evaluated visually from head maps showing electrodes contributing to the two independent components.

Associations with ERP measures were conducted for the specific language and literacy measures listed in Table 1. Missing data on child or parent behavioural tests were imputed by assigning a score equivalent to the individual from the same group with the closest average language test score. Predictions of four models of the relationship between Ta amplitude, language measures and parental language (Figure 1) were tested formally using structural equation modelling implemented with OpenMx software [41].

**Table 1:** Mean (SD) age, nonverbal IQ and scores on language/literacy tests in relation to age and SLI status.

Group	TD-child	TD-teen	SLI-child	SLI-teen
N male and female^1^	6 f 10 m	6 f 10 m	5 f 11 m	5 f 11 m
Age (yr)	9.75	13.80	9.82	14.06
	(1.29)	(1.06)	(1.27)	(1.20)
WASI PIQ	102.31	101.38	98.38	100.75
	(9.86)	(12.15)	(9.22)	(9.35)
Test for Reception of Grammar^2^	99.07	104.43	79.81	94.38
	(11.44)	(6.30)	(11.59)	(11.10)
NEPSY nonword repetition^3,4^	10.94	10.94	7.81	6.06
	(2.46)	(2.26)	(3.12)	(3.11)
NEPSY oromotor sequences^5^	0.25	0.03	−1.13	−0.94
	(1.00)	(1.08)	(0.81)	(1.19)
TOWRE phonetic decoding^2^	106.27	108.13	83.56	77.06
	(10.50)	(9.65)	(14.07)	(12.91)

1 Missing data: three on TROG-2, three on nonword repetition, three on oromotor sequences, and one on TOWRE;

2 Scaled with mean 100, SD 15;

3 Scaled with mean 10, SD 3;

4 Relative to own norms;

5 Five-point scale.

## Results

### Comparison of Mean Ta Amplitudes

**Figure 2:**
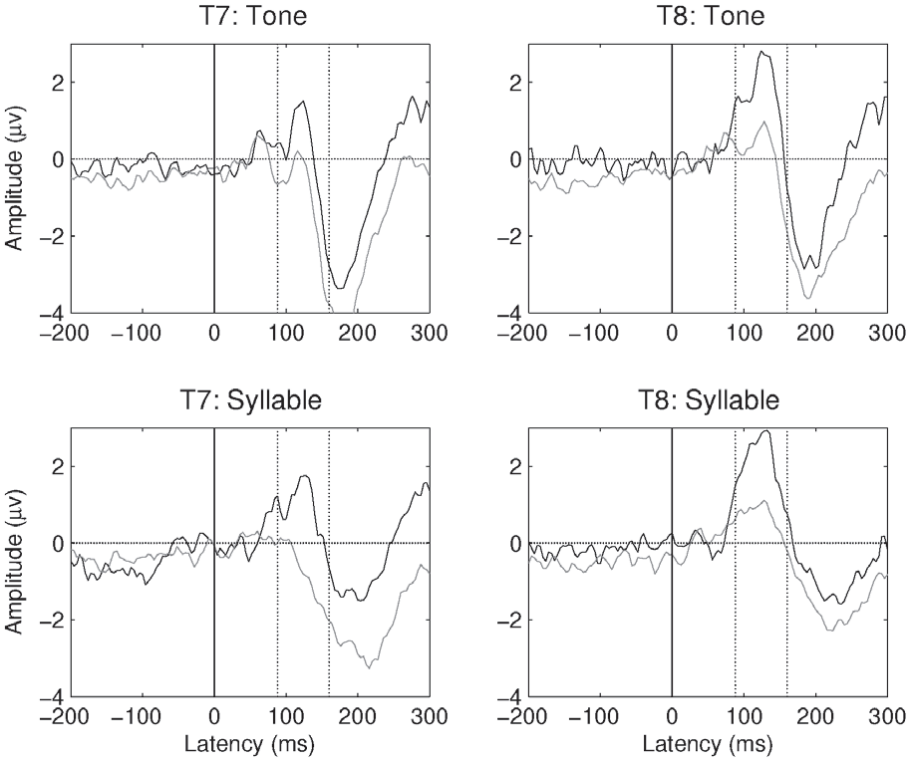
Average response at T7 and T8 for SLI (grey line) and TD (black line) children.

**Figure 3:**
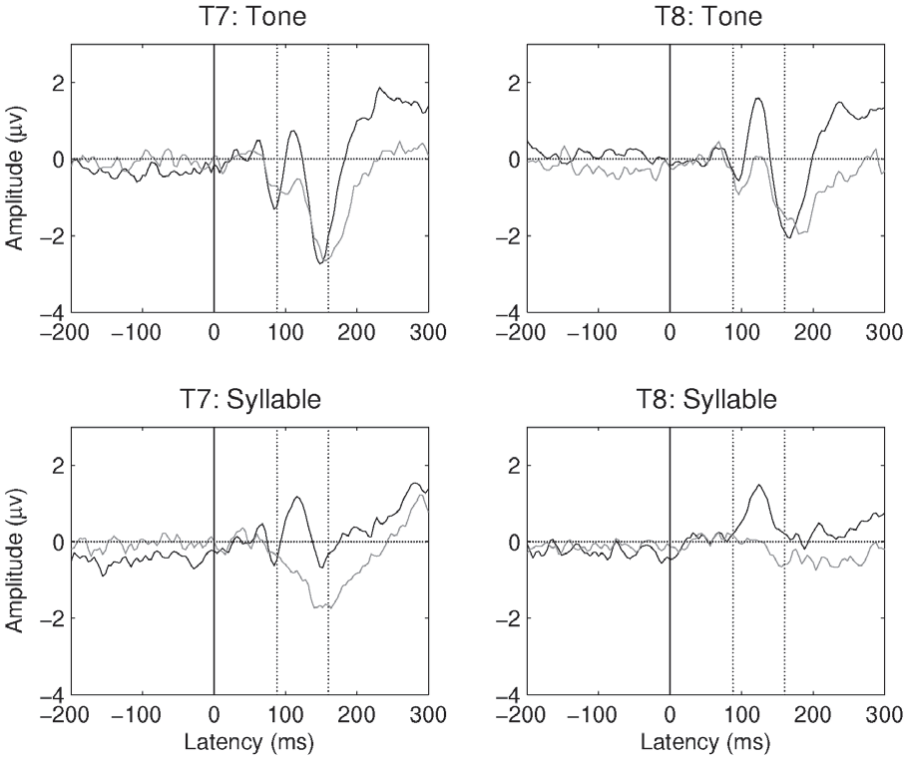
Average response at T7 and T8 for SLI (grey line) and TD (black line) teenagers.

Figures 2 and 3 show the grand average AERP for children and teens respectively, for tones and syllables, at temporal sites T7 and T8. Means are shown in Table 2. It is evident on inspection that the Ta is much less pronounced in the participants with SLI. ANOVA of mean amplitudes in the Ta region confirmed a significant main effect of SLI status, F (1, 60) = 17.7, p<.001, η^2^ = .23, as well as a significant effect of age band, F (1, 60) = 8.1, p = .006, η^2^ = .12, reflecting a decrease in Ta with age. As is evident from Figures 2 and 3, Ta was significantly larger at T8 (right temporal) than at T7 (left temporal), F (1, 60) = 54.6, p<.001, η^2^ = .48. This is noteworthy when one considers that stimulus presentation was monaural to the right ear, which might have been expected to lead to a greater left hemisphere response. There was also a significant effect of stimulus type, with a larger Ta for syllables, F (1, 60) = 16.8, p<.001, η^2^ = .22. No interactions were significant.

**Table 2:** Mean (SD) Ta amplitude for four groups by electrode and stimulus type.

Tones	Child TD	Child LI	Teen TD	Teen LI
T7	0.02 (1.42)	−0.96 (1.41)	−1.14 (1.06)	−1.35 (1.55)
T8	1.53 (1.16)	0.14 (1.03)	0.08 (0.97)	−0.52 (1.45)
Syllables				
T7	0.71 (1.73)	0.26 (1.84)	−0.79 (1.37)	−1.08 (1.36)
T8	2.05 (1.48)	0.83 (1.53)	0.69 (1.18)	−0.14 (1.09)

### Categorisation of Children According to Whether Ta was Present

For comparison with Shafer et al [19] participants were categorised according to whether or not they showed a Ta peak. The criterion for peak identification is rather arbitrary, but an attempt was made to set an objective cutoff, by using data from individual trials. At each time point, the set of amplitudes for all trials was compared with zero using a one-sample t-test. Given the large number of time-points, it is likely that some t-values will exceed conventional levels of significance by chance. A criterion was therefore identified that yielded a small number of ‘significant’ peaks in a portion of the waveform where no peaks were expected, i.e. end of the trial, from 724 to 800 ms post stimulus onset. The criterion used was different for tones, where the Ta was smaller and briefer in duration, than for syllables. For the tone condition, a peak was identified when there were at least two t-values greater than 2.32 (p<.01) in the time window; For 128 waveforms (64 participants×2 electrodes) this identified only six (5%) false ‘peaks’ in the 724–800 ms window. By contrast, in the Ta window of 88 to 160 ms, 57 (44%) significant peaks were identified by this criterion. For the syllable condition, the criterion was at least four t-values greater than 2.32 in the time window. This gave only two peaks (2%) in the 724–800 ms portion of the waveform, whereas 59 (46%) were detected in the Ta interval of 88–160 ms.

Table 3 shows the numbers of individuals with a significant Ta in the different stimulus conditions. The children and teens were added together for chi square analysis to test whether the distribution of the four categories of Ta occurrence differed for SLI and TD. For the tone condition, the difference fell short of significance: χ^2^ = 6.32, d.f. = 3, p = .10, but for the syllable condition, the difference was highly significant, χ^2^ = 14.7, d.f. = 3, p = .002. Nevertheless, consistent with the findings of Shafer et al [19], it was not unusual to find participants from the TD group who did not show a significant Ta on either left or right. Thus presence of a Ta peak would not be a useful diagnostic marker, despite the substantial group difference for the syllable condition.

**Table 3:** Number of individuals (out of 16) showing Ta in tone and syllable conditions.

Tones	Child TD	Child LI	Teen TD	Teen LI
No Ta	4	8	4	9
Ta on left only	0	1	1	0
Ta on right only	5	5	6	4
Ta left and right	7	2	5	3
Syllables				
No Ta	3	8	5	12
Ta on left only	0	1	2	0
Ta on right only	5	7	3	2
Ta left and right	8	0	6	2

Children in the SLI group had quite varied profiles of language difficulty, raising the possibility that an abnormal ERP might predict the language profile. To test this idea, a broad distinction was drawn between children with SLI who had no significant Ta at any electrode for either tones or syllables (N = 13) and the remainder (N = 19). Children were divided into those whose problems encompassed receptive language (defined as having a score more than 1 SD below the mean on either TROG or the ERRNI comprehension), and the remainder. The trend was for better comprehension in children who did *not* show a Ta, where 6 of 11 cases (46%) had a receptive language problem, compared with 11 of 19 (58%) of receptive problems in those who had at least one Ta. However, this difference was not reliable, Fisher exact test, p = .385.

### Comparison of Groups on Measure of Baseline Noise

A secondary analysis was conducted to determine whether the lower Ta values in SLI could be the consequence of noisier data, but this was not confirmed. ANOVA of the noise index (standard deviation during the baseline) revealed a significant effect of age, with noisier data in children than teens, but there was no effect of SLI (data not shown). Furthermore all correlations between Ta amplitude and baseline noise were very low and nonsignificant.

### Topography of Ta Responses

**Figure 4:**
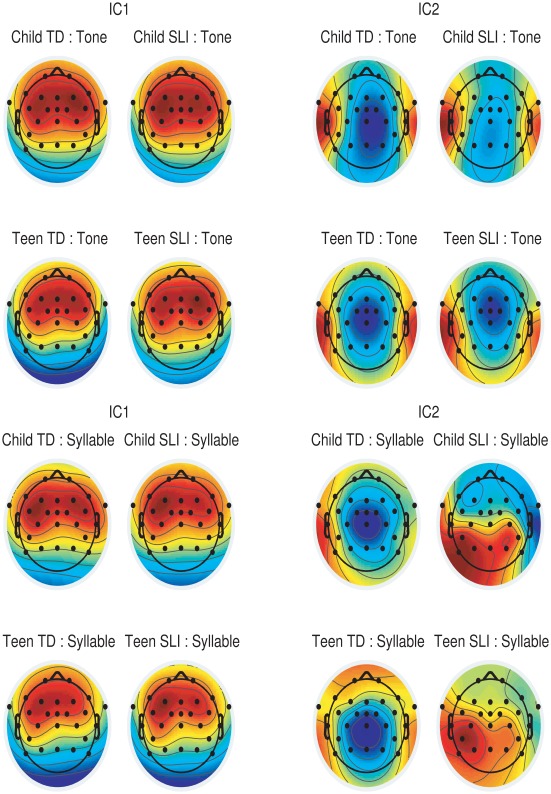
Weightings from electrodes for two independent components corresponding to the vertex and T-complex.

A further question concerned the spatial distribution of activity. Figure 4 shows grand average headplots for two independent components for the four groups in both conditions. Component 1 corresponds to the vertex response and component 2 to the T-complex. On inspection there is little evidence of any difference for the tone stimuli, but a marked difference for component 2 for syllables. Whereas the headplots are very similar for tones and syllables in the TD group, they are different for the SLI groups. In both children and teenagers, activity is far less focal for syllables than for tones, with asymmetric activity dominating left temporal and posterior sites.

### Correlations with Behavioural Measures

Table 4 shows the correlation matrix between Ta amplitudes and the selected language and literacy measures in children and their parents. Children show significant correlations between at least one Ta measure and nonword repetition, nonword reading and TROG. However, this does not tell us whether (i) abnormal brain responses lead to language problems (Endophenotype and Additive Risks models in Figure 1), (ii) language problems lead to abnormal brain responses (Neuroplasticity model), or (iii) a third factor increases the risk for both language problems and abnormal brain responses (Pleiotropy model). The familial risk measures, based on language and literacy test scores of parents, are useful for distinguishing causal accounts. As shown at the bottom of Table 4, all parent measures show significant correlations with at least two child language measures, but only one, oromotor skills, is significantly correlated with the child T-complex measures.

**Table 4:** Correlations between Ta and behavioural measures, whole sample (N = 64), with significant correlations bolded.

		1	2	3	4	5	6	7	8	9	10	11	12
1	Tone T7	1											
2	Tone T8	**.659**	1	.									
3	Syllable T7	.**625**	**.439**	1									
4	Syllable T8	**.359**	**.661**	**.560**	1								
Child													
5	TROG-2	.071	.034	.239	.084	1							
6	Nonword repetition	**.253**	**.321**	**.306**	**.374**	**.342**	1						
7	Nonword reading	.217	**.285**	**.357**	**.312**	**.409**	**.720**	1					
8	Oromotor	.175	.200	**.359**	**.270**	**.416**	**.637**	**.608**	1				
Parent													
9	TROG-2	.142	.123	.076	-.001	.051	**.326**	**.373**	.153	1			
10	Nonword repetition	.131	.139	.058	.119	.183	.208	.**314**	.092	**.321**	1		
11	Nonword reading	.105	.129	.111	.094	.179	**.325**	**.398**	.186	**.403**	**.603**	1	
12	Oromotor	**.295**	**.382**	**.302**	**.407**	.15	**.311**	**.443**	**.305**	.179	**.576**	**.571**	1
	Mean value	−0.86	0.31	−0.23	0.85	−0.40	−0.35	−0.43	−0.38	−0.16	−0.51	−0.42	−0.60
	SD	1.44	1.37	1.72	1.52	0.92	1.14	1.20	1.19	0.99	1.28	1.26	1.12

Means and SDs below.

Note, significance levels: r = .25, p<.05; r = .32, p<.01; r = .41, p<.001.

### Maximum Likelihood Testing of Structural Models

To relate these data to the models in Figure 1, we specified latent variables of Auditory deficit and Language impairment. Initially, the indicators of the Auditory deficit variable were the four measures of Ta amplitude (tones and syllables, at T7 and T8). However, these gave a poor-fitting model, because the Tone T7 measure was weakly correlated with the Syllable T8 measure. Dropping Tone T7 and equating paths from the three other indicators to the Auditory factor gave a good factor structure fit which was therefore used in the final causal models. The Language impairment latent variable was indicated by the four language/literacy measures. The Family risk variable was indicated by the corresponding four language/literacy measures from parents. Before proceeding to formal fit of the models, we can consider how well each agrees with the data, simply by comparing the pattern of correlations between variables, using path-tracing rules to estimate expected values [42]. Consider the Endophenotype model (Figure 1). If x is the path from Family Risk to Auditory Deficit and y is the path from Auditory Deficit to Language Impairment, then z, the Path from Family Risk to Language Impairment = x.y. Thus if correlation between Family Risk and Language Impairment (x) is negligible, we should see no association between Auditory Deficit and Language Impairment. This follows because the Endophenotype model regards Auditory Deficit as a mediating variable that accounts for the association between parent and child language. This prediction does not appear to be supported by the data in Table 4, which show stronger correlations between parent and child language measures than between parent measures and child T-complex measures.

The Additive Risks model is more plausible, because it predicts that x, the correlation between Family Risk and Auditory Deficit, will be zero, broadly consistent with observed data. According to this model, Auditory Deficit serves to moderate the impact of genetic risk, but it is not caused by genetic risk. The Pleiotropy model fares less well, as it predicts that y = x.z, i.e. once again, a zero correlation between Family Risk and Language Impairment would entail that there would be no significant correlation between Auditory Deficit and Language Impairment. Finally, the Neuroplasticity model predicts that x = y.z. A low and nonsignificant value of x would be consistent with this model, provided that y and/or z were not large.

These informal impressions of model plausibility can be subjected to more formal evaluation using structural equation modeling to estimate goodness of fit. The current sample size is smaller than is usually recommended for such analysis, but given the simplicity of the models, with covariance between just three latent variables to be explained, we felt justified in carrying out a comparison of different model fits. The four models in Figure 1 were tested for fit against a baseline (“saturated”) model that included the three latent variables and their indicators, plus covariances between all three latent variables. Fit indices are shown in Table 5; a model with good fit will have a non-significant chi square value, indicating that the observed covariances are consistent with those predicted by the model. The Akaike Information Criterion (AIC), and RMSEA (Root Mean Square Error of Approximation) can be used to compare models, with lower values indicating better fit, whereas for the Cumulative Fit Index (CFI), a higher value corresponds to better fit. In addition, models can be compared with the fit of the saturated model, in which all covariances between latent variables are included. If the new model does not differ significantly from the saturated model, this indicates that the paths that differentiate the two models can be dropped without affecting model fit.

**Table 5:** Model fit and unstandardised path estimates (SE) for models from Figure 1.

Model	Endophenotype	Additive Risks	Pleiotropy	Neuroplasticity
χ^2^	55.1	51.7	53.5	48.0
d.f.	44	44	44	44
P	.121	.197	.150	.312
AIC	−32.9	−36.3	−34.5	−40.0
CFI:	.994	.995	.995	.996
RMSEA:	.102	.094	.098	.084
**Change in fit relative to saturated model**				
χ^2^	7.38	3.99	5.75	0.29
d.f.	1	1	1	1
P	.006	.046	.017	.590
**Paths between latent variables**				
Fam->Aud	0.40 (0.19)	–	0.42 (0.19)	–
Fam->Lang	–	0.53 (0.19)	0.65 (0.20)	0.60 (0.19)
Aud->Lang	0.55 (0.17)	0.48 (0.19)	–	–
Lang->Aud	–	–	–	0.47 (0.16)
**Paths from Child Language**				
TROG	0.36 (0.10)	0.33 (0.09)	0.35 (0.10)	0.36 (0.10)
Nonword repetition	0.83 (0.12)	0.75 (0.11)	0.79 (0.12)	0.82 (0.12)
Nonword reading	0.87 (0.12)	0.82 (0.11)	0.88 (0.12)	0.89 (0.12)
Oromotor	0.76 (0.13)	0.69 (0.11)	0.72 (0.13)	0.74 (0.13)
**Paths from Parent Language**				
TROG	0.40 (0.13)	0.43 (0.13)	0.41 (0.13)	0.43 (0.13)
Nonword repetition	0.98 (0.15)	0.96 (0.16)	0.94 (0.15)	0.96 (0.15)
Nonword reading	0.98 (0.15)	1.04 (0.15)	0.97 (0.15)	1.02 (0.15)
Oromotor	0.85 (0.14)	0.79 (0.13)	0.86 (0.14)	0.82 (0.14)
**Paths from Auditory (constrained to be equal)**				
Tone T8	1.06 (0.13)	1.15 (0.13)	1.06 (0.13)	1.00 (0.13)
Syllable T7	1.06 (0.13)	1.15 (0.13)	1.06 (0.13)	1.00 (0.13)
Syllable T8	1.06 (0.13)	1.15 (0.13)	1.06 (0.13)	1.00 (0.13)

**Figure 5:**
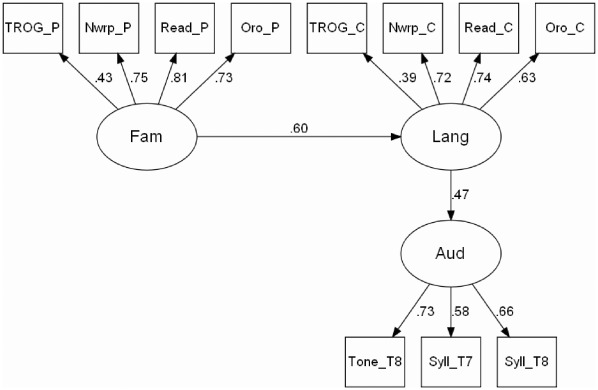
Neuroplasticity model specification for structural equation modeling, with estimated values for standardised paths. N.B. estimated variances omitted from diagram; variances for latent variables fixed at 1.0. Key: _P = parent measure; _C = child measure; Nwrp = Nonword repetition; Read = Nonword reading; Oro = Oromotor sequences; Fam = Family risk; Lang = Language impairment; Aud = Auditory deficit.

Satisfactory fit was obtained for all four models, but the only model that gave as good a fit as the saturated model was the Neuroplasticity model, in which the causal relationship goes *from* Language Impairment *to* Auditory Deficit. Estimated standardized paths for this model are shown in Figure 5. These allow direct comparison of effect sizes for different paths, whereas the unstandardized paths shown in Table 5 are scaled in the original units of measurement.

## Discussion

We replicated the study of Shafer et al [19], finding robust differences between SLI and TD groups in the amplitude of the Ta, particularly when syllables were used. Our findings demonstrate abnormal responsiveness to meaningless sounds at an early stage of cortical processing in a passive task requiring no explicit discrimination. Significant group differences were also seen for pure tones, indicating that the impairment is not specific to speech.

As in Shafer et al. [19], discrimination between the TD and SLI groups was far from perfect when a categorical classification of T-complex was used. Even though the effect size of the difference between TD and SLI children was relatively large by the standards of this field, many of the TD children did not show a T-complex, whereas some children with SLI did. It would not, therefore, be possible to use the T-complex as a diagnostic measure for SLI.

Our stimuli were presented monaurally to the right ear. Nevertheless, as in previous studies, we found that the T-complex was greater on the right, ipsilateral side. This effect was seen in all groups, however, and did not interact with SLI/TD status. The fact that the abnormality of the T-complex was just as pronounced in teenagers with SLI as in children speaks against any simple explanation in terms of delayed maturation, although it would be compatible with a model such as that proposed by Wright and Zecker [27], who proposed that maturation of auditory function is terminated at puberty.

The analysis of variability in the baseline period allowed us to rule out an explanation in terms of noisier ERPs in the participants with SLI. Scrutiny of the headplots of two independent components in the Ta time period revealed, however, more topographically diffuse activity in children and teenagers with SLI when syllables were used. This finding is compatible with an earlier study of the same dataset in which we analysed late-discriminative negativity responses to stimulus change in series of tones or syllables [30]. In that study, the evidence was more indirect, but on the basis of an analysis of synchronisation of brain responses across different epochs, we proposed that analysis of sound characteristics may involve activation in a broader region of cortex in those with SLI.

A significant relationship was found between the size of the Ta component and some of the language measures. Such findings are often interpreted as evidence that language impairment is the consequence of an auditory deficit that is evident in the ERP, and which itself is caused by some earlier causal factor such as genetic risk. This causal model, which we term the Endophenotype model, was the least well-fitting of the models that we considered. Our data support the notion that SLI is heritable: there were significant correlations between parent and child language measures. However, the failure to find significant correlations between most measures of parent language status and child auditory ERP deficit does not agree with the notion that auditory deficit is the mediating factor leading to this parent-child correlation.

The best fit was obtained with the Neuroplasticity model; this regards abnormalities of the ERP as a *consequence* of language impairment. Thus a genetic risk factor may affect the child’s ability to form phonological categories, which has a knock-on effect on the representation of sound in the brain as reflected in the ERP. It would be rash to assume this model is the only explanation for the results; the sample size is relatively small for structural equation modeling, and other models, which assumed the reverse direction of causation, achieved an acceptable fit. Furthermore, the test of oromotor skills showed a different pattern of correlations from other indicators of family risk, with significant correlations between parental scores and child Ta. Given the small sample size and the failure to find this pattern for other heritable language measures, we cannot rule out sampling error as an explanation, but this result does suggest that speech production may be more strongly linked to the auditory ERP than those that tax phonological categorisation or memory.

Further indirect support for a Neuroplasticity model comes from the categorical analysis that suggested that the Ta abnormality was more marked for syllable stimuli than for pure tones. We know that phoneme-specific representations develop in the brain on the basis of experience with sounds of a language [43]–[44]. Furthermore, mismatch responses to sound changes in a second language can be affected by exposure to that language [45], and differ in bilinguals according to their proficiency in detecting those changes [46]. It is thus feasible that poor phonological skill, arising for whatever reason, might lead to development of less focal phonemic representations. It would be possible to test this idea by repeating the study with different stimuli in individuals with normal language skills: One would predict a broader topographical distribution of T-complex activity for non-native than for native sounds.

In sum, in a field where many findings are inconsistent or inconclusive, the finding of robust differences in the AERP at temporal sites is striking. The waveforms obtained here are remarkably similar to those reported by Shafer et al [19], despite differences in stimuli and technical details such as reference electrode. However, optimism that this is an ERP signature for SLI that could be used for early diagnosis is not justified, given the large numbers of typical children who had no Ta. In addition, the Ta does not appear to be a viable endophenotype for genetic studies. The causal analysis suggests that a small and non-focal Ta peak appears more likely to be the consequence of poor phonological skills than an underlying cause of SLI. The results are compatible with the idea that development of perceptual representations in the child’s brain is affected by expertise and experience.
